# Editorial: Appraisal processes in moral judgment: resolving moral issues through cognition and emotion

**DOI:** 10.3389/fpsyg.2023.1233865

**Published:** 2023-06-20

**Authors:** Justin F. Landy, Tom R. Kupfer

**Affiliations:** ^1^Department of Psychology and Neuroscience, Nova Southeastern University, Fort Lauderdale, FL, United States; ^2^School of Social Sciences, Nottingham Trent University, Nottingham, United Kingdom

**Keywords:** appraisal, moral judgment, emotion, cognition, context

The earliest work in moral psychology viewed moral judgment as dependent on our capacities to reason, which grew along with cognitive development (Piaget, 1965; Kohlberg, 1971). More recent influential accounts argued for the centrality of emotions in arriving at moral judgment (Greene et al., 2001; Haidt, 2001). According to Haidt's Social Intuitionist Model, brief feelings such as disgust drive our moral judgments whereas the role of reasoning is primarily to justify those judgments. Greene and colleagues argued for a Dual Process Model of morality in which a fast, automatic, and emotional route tends to lead to deontological moral judgment, whereas slower, more deliberative reasoning is required to arrive at utilitarian judgments. The ensuing two decades have seen vigorous debate between advocates of emotional primacy (e.g., Haidt and Bjorklund, 2008) and rational primacy (e.g., Royzman et al., 2014).

This controversy in moral psychology was foreshadowed by an earlier debate concerning the roles of cognition and emotion in judgment and decision making (Lazarus, 1999). That debate was resolved, in part, by introducing the concept of appraisal, which helped explain the ways in which emotions are connected to cognitive processes (Giner-Sorolla, 2019). Moral emotions and moral judgment may also depend on how people appraise specific aspects of a situation (Giner-Sorolla et al., 2018), and these appraisals can vary between people, leading to varying emotions and judgment (Kuppens et al., 2007). The same moral wrongdoing can be judged quite differently depending on an observer's appraisal of factors such as their relationship to the victim and perpetrator (Bloom, 2011; Earp et al., 2021), or their appraisal of cues to the importance of impartiality (McManus et al., 2020). Research into such contextual features, and the appraisal processes that connect them to moral emotions and moral judgment, has the potential to advance the field of moral psychology beyond debates over the primacy of cognition or emotion.

The importance of contextual factors in driving moral emotions and cognition, and also moral judgments and real behaviors, is a major recurring theme in this Research Topic. Taken together, the papers in this Research Topic illustrate that how we appraise moral situations depends greatly on the specifics of the situation, not just our more abstract, decontextualized moral beliefs and values.

Van den Berg et al. take up this issue at the theoretical level. They develop a conceptual model of how general moral values could result in moral behavior (or not), that is meant to be acceptable to both rationalist and intuitionist researchers. Based on this model, they predict that general moral values should be poor predictors of specific, contextualized moral behaviors, because there are a multitude of opportunities for contextual factors to moderate or overwhelm the direct effect of such values. They generally find support for this prediction across two measures of general moral values (Moral Foundations Questionnaire and Morality as Cooperation Questionnaire), and three types of moral behaviors (volunteering, adhering to COVID-19 restrictions, refraining from eating meat). They also find that a more specific, contextualized value (animal welfare) is a somewhat stronger predictor of relevant behavior (refraining from eating meat). They argue that “morality's influence on behavior may be more context specific than a general questionnaire can grasp” (p. 14). However, the authors do not *directly* investigate such contextual influences in the present work.

Zhang et al. investigate the effect of one contextual factor (psychological distance) on spontaneous inferences of (in)justice, drawing on Construal Level Theory for theoretical grounding. Using a probe recognition task, they find behavioral signatures of spontaneous justice inferences from actions that are psychologically close (in space or time) and actions that are psychologically distant, but such inferences appear to be stronger when actions are psychologically distant. In other words, the abstract concept of justice is activated more strongly for acts occurring thousands of miles away, compared to acts occurring a few miles away, and for acts occurring in the far future, compared to acts occurring in the near future. This is consistent with Construal Level Theory, which says that psychologically distant events activate more abstract, high-level cognition, and it illustrates one way in which context can affect implicit processes involved in appraising the moral characteristics of a situation.

Navarick and Moreno investigate whether a context that demands impartiality in decision-making (a hospital) eliminates previously observed biases in decisions about which of two individuals to help (e.g., kinship bias, age bias). While they generally find that such biases persist in this context, the picture is more nuanced than that. In particular, the biases often disappear or even reverse as the stakes get lower. For instance, their participants rate an 8-year-old as more deserving of treatment, and rate themselves as more likely to choose her for treatment, than an 80-year-old in a life-or-death situation. However, when the situation is less extreme, and the risk is injury due to falling, this bias reduces or reverses. So, we again see that context matters, in quite nuanced ways. The impartial context of a hospital does not *necessarily* lead to impartial judgments and action intentions, but it can sometimes do so, depending on the probable outcomes.

Lastly, Ye et al. examine predictive relationships among tourists' and employees' environmentally responsible behaviors at three forested tourist destinations. They find that tourists report that witnessing employees engage in pro-environment behaviors increases the likelihood of engaging in such behaviors themselves. This contagion effect is mediated by two facets of moral elevation: elevating emotions and positive views of humanity. Interestingly, the effect via elevating emotions is moderated by environmental knowledge, such that it is stronger among tourists with more knowledge, an example of how emotion and cognition interact to give rise to moral behaviors. Here again we see a contextual influence on morality (in this case, moral behavior): engaging in pro-environmental behaviors is driven, at least in part, by witnessing others do so, not exclusively by abstract, pro-environmental values.

Overall, the papers in this Research Topic demonstrate the utility of attending to contextual influences in the study of appraisal processes in morality. Future work aimed at explaining and accounting for moral emotions, cognition, judgments, and behaviors must account for the often-complex influences of contextual factors. We see the current Research Topic as a promising step in this direction.

## Author contributions

JL and TK drafted, revised, and approved the manuscript. All authors contributed to the article and approved the submitted version.

## References

[B1] Bloom P. (2011). Family, community, trolley problems, and the crisis in moral psychology. Yale Rev. 99, 26–43. 10.1353/tyr.2011.006127885969

[B2] Earp B. D. McLoughlin K. L. Monrad J. T. Clark M. S. Crockett M. J. (2021). How social relationships shape moral wrongness judgments. Nat. Commun. 12, 5776. 10.1038/s41467-021-26067-4 34599174 PMC8486868

[B3] Giner-Sorolla R. (2019). The past thirty years of emotion research: appraisal and beyond. Cogn. Emot. 33, 48–54. 10.1080/02699931.2018.1523138 30221581

[B4] Giner-Sorolla R. Kupfer T. Sabo J. (2018). “What makes moral disgust special? An integrative functional review,” in Advances in Experimental Social Psychology. Cambridge, MA: Academic Press. p. 223–289. 10.1016/bs.aesp.2017.10.001

[B5] Greene J. D. Sommerville R. B. Nystrom L. E. Darley J. M. Cohen J. D. (2001). An fMRI investigation of emotional engagement in moral judgment. Science. 293, 2105–2108. 10.1126/science.1062872 11557895

[B6] Haidt J. (2001). The emotional dog and its rational tail: a social intuitionist approach to moral judgment. Psychol. Rev. 108, 814–834. 10.1037/0033-295X.108.4.814 11699120

[B7] Haidt J. Bjorklund F. (2008). “Social intuitionists answer six questions about morality,” in Moral Psychology Vol. 2, ed W. Sinnott-Armstrong (MIT Press).

[B8] Kohlberg,. L. (1971). “From is to ought: How to commit the naturalistic fallacy and get away with it in the study of moral development,” *Cognitive Development and Epistemology*, ed T. Mischel (New York, NY: Academic Press), 151–235. 10.1016/B978-0-12-498640-4.50011-1

[B9] Kuppens P. Van Mechelen I. Smits D. J. De Boeck P. Ceulemans E. (2007). Individual differences in patterns of appraisal and anger experience. Cogn. Emot. 21, 689–713. 10.1080/02699930600859219

[B10] Lazarus R. S. (1999). The cognition-emotion debate: A bit of history. In T. Dalgleish & M. J. Power (Eds.), *Handbook of cognition and emotion* (pp. 3–19). Chichester, UK: Wiley 10.1002/0470013494.ch1

[B11] McManus R. M. Kleiman-Weiner M. Young L. (2020). What we owe to family: the impact of special obligations on moral judgment. Psychol. Sci. 31, 227–242. 10.1177/0956797619900321 31990627

[B12] Piaget J. (1965). The Moral Judgment of the Child. New York: Free Press.

[B13] Royzman E. B. Landy J. F. Goodwin G. P. (2014). Are good reasoners more incest-friendly? Trait cognitive reflection predicts selective moralization in a sample of American adults. Judgm. Deci. Mak. 9, 176–190. 10.1017/S1930297500005738

